# A Novel CFA3 Locus Encompassing *KCNIP4* Is Associated with Idiopathic Epilepsy in Siberian Huskies

**DOI:** 10.3390/genes17040459

**Published:** 2026-04-15

**Authors:** Tracy A. Smith, Leon Potisk

**Affiliations:** Department of Biological Sciences, University of Maryland, Baltimore County, 1000 Hilltop Circle, Baltimore, MD 21250, USA; spotisk1@umbc.edu

**Keywords:** Siberian Huskies, idiopathic epilepsy, primary epilepsy, GWAS, *KCNIP4*

## Abstract

*Background/Objectives:* Idiopathic epilepsy is a lifelong neurologic disorder in dogs, but its genetic basis remains incompletely understood in many breeds. This study aimed to identify risk-associated markers in Siberian Huskies, quantify their effects, assess potential risk modifiers, and characterize the shared haplotype background of the associated signal. *Methods:* A genome-wide association study was conducted in 113 Siberian Huskies genotyped on the Illumina CanineHD array, integrating association, regression, and haplotype/IBD analyses. An independent follow-up cohort of 57 additional dogs was genotyped at the lead marker by Sanger sequencing. Sex and gonadectomy status/timing were also evaluated as potential modifiers of risk, using multivariable regression and time-to-event analyses. *Results:* A strong, localized association was identified on canine chromosome 3 (CFA3) within *KCNIP4*. The lead intronic marker was significantly enriched in cases, with all risk-allele homozygotes affected, most heterozygotes affected, and no control homozygotes observed. Risk-associated chromosomes shared extended haplotypes across the region, consistent with carriers inheriting a common risk haplotype from a relatively recent shared ancestor. Among carriers, male sex was associated with higher odds of epilepsy and earlier seizure onset, with more tentative evidence for a similar association with gonadectomy before 5 years of age. *Conclusions:* These findings prioritize a CFA3 region encompassing *KCNIP4* as a major risk locus for idiopathic epilepsy in Siberian Huskies. Fine-mapping with high-coverage sequencing and functional follow-up will be required to pinpoint the causal variant(s) and support development of risk assessment tools. Until those studies are completed, this marker should be regarded as a research finding rather than a predictive test.

## 1. Introduction

Idiopathic epilepsy (IE) is one of the most common chronic neurological disorders in dogs, often requiring lifelong management and imposing substantial welfare, emotional, and financial burdens on affected dogs and their owners [1,2,3]. In general dog populations, reported prevalence estimates for epilepsy are approximately 0.6–0.8% [4]. In Huskies, prevalence has been estimated at 0.67% (95% CI 0.42–0.92) [5], although it is unclear whether this estimate includes all Husky-type dogs (e.g., Alaskan Huskies) or Siberian Huskies only. Although robust breed-specific prevalence estimates in Siberian Huskies are limited, multiple lines of evidence—including large multibreed datasets [5,6], insurance claims [7], and consistent reports from breeders, breed clubs [8], and veterinarians—indicate that IE occurs at a measurable rate in the breed and represents an important health concern.

The genetic basis of IE in dogs has been the focus of sustained investigation [9,10,11,12,13,14,15,16,17,18]. Across breeds, *ADAM23,* a gene on chromosome 37 (CFA 37) involved in synaptic excitability and orthologous to human *ADAM23*, represents one of the most consistently replicated common-risk loci, supported as a low-penetrance susceptibility factor in multiple breeds [12]. Beyond *ADAM23*, additional gene discoveries in various breeds highlight the genetic and phenotypic heterogeneity of canine epilepsy [9,10,11,15,16], and these findings emphasize not only the complexity of epilepsy genetics in dogs but also the importance of breed-specific investigations to capture diverse etiologies.

Arctic dogs comprise an evolutionarily distinct canid lineage that arose by the end of the Pleistocene and was firmly established by the early Holocene [19,20]. This deep demographic history, and subsequent diversification under intense selection for function and regional adaptation, suggests they may carry distinct genetic risk variants for traits such as idiopathic epilepsy. Importantly, Siberian Huskies are not genetically homogeneous: recent genomic analyses support at least four differentiated subpopulations within the breed (show, pet, racing, and Seppala), reflecting partially distinct breeding histories, patterns of gene flow [19] and selection on different traits [21]. Accounting for this within-breed structure is therefore essential for both locus discovery and interpretation, because true risk haplotypes may be unevenly distributed across subpopulations and because residual stratification can inflate association signals if not explicitly addressed.

Here, a genome-wide association approach was used to identify loci associated with idiopathic epilepsy in Siberian Huskies while accounting for relatedness and lineage structure. Association signals were identified using mixed models incorporating a kinship matrix and principal components, together with complementary lineage-stratified sensitivity analyses and follow-up evaluation of the lead marker in an independent Sanger-genotyped cohort. The implicated interval was then further characterized using haplotype and IBD analyses to map the shared risk chromosome around the signal and to estimate how recently the risk haplotype spread through the population.

## 2. Materials and Methods

### 2.1. Sample Collection and Phenotyping

Participants were recruited through breed networks and social media, and owners contributed Embark raw genotype files (Embark Veterinary Inc., Boston, MA, USA) for this discovery phase. The discovery cohort comprised 113 Siberian Huskies (63 males, 50 females), including 111 purebreds and two mixed-breed dogs with ≥50% Siberian Husky ancestry (Supplementary Table S1). This dataset included 24 dogs with suspected idiopathic epilepsy (16 males and 8 females) and 89 neurologically healthy controls (47 males and 42 females). To minimize phenotype misclassification, controls were required to be seizure-free at enrollment and at least 7.5 years old. Case status was supported by veterinary medical records and owner-provided histories, with affected dogs meeting International Veterinary Epilepsy Task Force (IVETF) Tier I criteria, defined as at least two unprovoked seizures occurring more than 24 h apart, age at onset between 6 months and 6 years, an unremarkable interictal physical and neurologic examination, and no significant abnormalities on routine bloodwork [22]. Dogs with a history suggesting a reactive cause or structural brain disease (e.g., significant head trauma, cancer/neoplasia, or infectious/inflammatory disease such as Lyme and zinc-responsive dermatosis) were excluded to reduce confounding by systemic or acquired conditions that can cause seizures or mimic epilepsy in this breed.

To augment the dataset and permit follow-up evaluation of the association, we assembled an additional “Sanger cohort” of 73 Siberian Huskies (57 unique dogs: 24 cases, 33 controls) from owner-submitted buccal swabs and samples obtained through the OFA/CHIC DNA repository (Supplementary Table S2). Sixteen of the 73 dogs overlapped the discovery cohort and were used to confirm concordance with the array-based genotypes but deduplicated for downstream analyses. The same case/control criteria were applied, with cases allowing seizure onset up to 9 years of age. These dogs underwent Sanger sequencing of a 574-bp interval flanking the lead GWAS SNP. The final pooled dataset comprised 170 dogs, including 48 cases (32 males and 16 females) and 122 controls (62 males and 60 females).

All dog owners provided written or electronic informed consent prior to enrollment and all genotype files, buccal swabs, and metadata were submitted voluntarily. All work involving canine-derived data was reviewed and approved by the University of Maryland, Baltimore County (UMBC) Institutional Biosafety Committee (IBC; protocol #1937).

### 2.2. Genotyping and Quality Control

All dogs in the discovery cohort were genotyped on the Illumina CanineHD array (236,780 SNPs) as customized by Embark Veterinary, Inc. Genotypes were processed in PLINK v1.9 [23,24]. Variants were excluded if they were missing genotype calls in more than 10% of samples or minor allele frequency (MAF) was <0.01. After quality control, the final discovery dataset contained 153,704 variants and 113 dogs with a genotyping rate of 0.961. Variant positions refer to the CanFam3.1 reference genome [25].

Buccal swabs collected for the Sanger cohort were extracted using either the QIAamp DNA Mini Kit (QIAGEN, Hilden, Germany) or an ethanol precipitation workflow using PG-L2P (DNA Genotek Inc., Ottawa, ON, Canada), following manufacturer-recommended protocols. Target amplicons were generated by PCR in 50 µL reactions comprising 25 µL DreamTaq Hot Start PCR Master Mix (Thermo Fisher Scientific, Waltham, MA, USA), forward 5′-TGTCCTATGCTCCCATTCTTTG and reverse 5′-CTCAGATCCTGTCTCAGCTACT primers (1–2.5 µL each), template DNA (5 µL), and nuclease-free water to volume. Thermal cycling was performed with an initial denaturation at 95 °C for 3 min, followed by 40 cycles of 95 °C for 30 s, primer-specific annealing at 55 °C for 30 s, and 72 °C for 60 s, with a final extension at 72 °C for 10 s. Unpurified PCR products were submitted to Azenta Life Sciences/GENEWIZ (South Plainfield, NJ, USA) for standard Sanger sequencing.

### 2.3. Genome-Wide Association Analysis and Dosage-Based Risk Modeling

For downstream structure and kinship analyses, a linkage disequilibrium (LD) pruned marker set was generated in PLINK v1.9 (--indep-pairwise 50 5 0.5) and used to compute principal components (PCs) and a genomic relatedness matrix (GRM). Including PCs and relatedness as fixed effects is particularly important in dog datasets because population structure is common even within a single breed (e.g., line/subpopulation differences, geographic substructure, and/or recent admixture), and these ancestry gradients can confound association signals if not modeled explicitly. Genome-wide association testing was performed in GEMMA v0.98.5 [26] using a univariate linear mixed model (lmm) with a centered kinship matrix (-gk 1) to control for cryptic relatedness. A baseline mixed model was fit including the GRM and no fixed-effect covariates, and additional mixed models were fit that included principal components as fixed effects. Sensitivity to residual structure was evaluated by comparing models adjusted for PC1–PC2 versus expanded adjustment (PC1–PC5), since higher-order PCs can capture additional, subtler ancestry differences and subpopulation stratification that may not be fully accounted for by the GRM and the leading PCs alone. Consistent results across these adjustments increase confidence that association signals are not driven by unmodeled population structure. Unadjusted allelic association testing was performed in PLINK v1.9 (--assoc) [23,24] to obtain allelic odds ratios and case/control allele frequencies.

Next, dogs were classified into two main owner-reported lineage groups defined by phylogenetic clustering [19]: Show/Pet (including Pet, Show, Sled-Show, and Husky Mix) and Working (Racing and Seppala). The GEMMA linear mixed-model association analysis was then repeated within the Show/Pet subset alone (*n* = 58, 17 cases) to evaluate whether the CFA3 signal persisted within a more homogeneous lineage background, since nearly all cases carrying the lead risk-associated marker were from the Show/Pet group with only one case carrying the lead marker from the Working group (Supplementary Table S1). The same lmm framework was used, including the kinship matrix and principal components (up to PC1–PC5) as fixed-effect covariates.

Genomic inflation (λGC), defined as the ratio of the observed median chi-square test statistic to the expected median under the null, was calculated from the GEMMA mixed-model Wald *p*-values to assess residual test-statistic inflation, comparing models without PC covariates to models including PCs as fixed effects. Multiple testing was controlled using (i) a genome-wide Bonferroni threshold of *p* < 3.25 × 10^−7^ (0.05/153,704 SNPs) and (ii) the Benjamini–Hochberg false discovery rate (FDR) procedure computed in R v4.1.2 using p.adjust with method = “fdr” [27]. Genome-wide significance was defined using the Bonferroni-corrected threshold as the primary criterion for inference, while FDR-adjusted values were reported as complementary measures to summarize the broader pattern of association under a less conservative multiple-testing framework. To further control for lineage structure, association testing was repeated in PLINK using a Cochran–Mantel–Haenszel test (--mh), stratifying by owner-reported lineage, and potential effect heterogeneity across strata was evaluated with a Breslow–Day test (--bd). To determine whether the CFA3 association reflected a single primary signal or multiple independent effects, a conditional mixed-model GWAS was performed in GEMMA by adding lead-SNP dosage (0/1/2 copies of the risk allele) as a fixed-effect covariate (with PC1–PC2 retained); disappearance of the regional peak after conditioning was interpreted as evidence for a single main signal tagged by the lead variant. Manhattan and quantile–quantile (QQ) plots were generated in R v4.1.2 using qqman v0.1.8 [28].

To quantify dose-dependent effects of the CFA3 lead associated marker (BICF2P1282800) on epilepsy risk, the GWAS discovery and Sanger follow-up cohorts were pooled (*n* = 170), and an additive logistic regression model was fit with epilepsy case/control status as the outcome and risk-allele dosage (0, 1, or 2 copies) as the predictor, yielding a per-allele log-odds effect (β) and corresponding odds ratio (OR = exp[β]). Because no seizure-free controls were homozygous for the risk allele (although heterozygous controls were present), standard logistic regression was prone to instability; therefore, Firth-penalized logistic regression was used as the primary estimator to reduce bias and provide stable inference as implemented in the R package logistf v1.26.1 [29]. For the Firth model, *p*-values were taken from the penalized-likelihood inference provided by logistf. For comparison, standard maximum-likelihood logistic regression (binomial GLM) was also fit as a sensitivity analysis to test whether the estimated odds ratio and its significance were robust to model choice.

### 2.4. Age-at-Onset Distribution and Modifier Analyses in CFA3 Risk-Allele Carriers

We first characterized age distribution of epilepsy onset among CFA3 risk-allele cases with at least one copy of the risk allele at the lead marker, BICF2P1282800, in the pooled dataset by summarizing age at first seizure using nonparametric descriptive statistics. Median age at onset and the interquartile range (IQR; 25th–75th percentile) were stratified by sex.

This summary informed two distinct age landmarks used in our analyses for seizure-free controls. First, because nearly all observed seizure onsets occurred by 5 years of age, controls were required to be at least 7.5 years old to ensure they had passed the typical age-of-onset window. Although two carriers had later onset, this threshold substantially reduced the risk of including dogs as controls before they had fully aged through the period of greatest susceptibility, without unnecessarily sacrificing sample size. Second, we defined gonadal status at a 5-year landmark to capture endogenous hormone exposure during the cohort’s highest-susceptibility period (juvenile through early adult), when sex steroids are plausibly linked to neuronal excitability and seizure threshold [30], while de-emphasizing hormonal changes occurring after the onset window had largely diminished.

Next, we analyzed dogs carrying at least one copy of the CFA3 risk allele in the pooled dataset (*n* = 36) to evaluate whether sex and gonadal status were associated with epilepsy status among carriers. Epilepsy status (case vs. seizure-free control) was modeled as a binary outcome using Firth penalized logistic regression to accommodate sparse cell counts and mitigate separation. Predictors included sex (male vs. female) and gonadal status (neutered/spayed vs. intact). Age at gonadal removal (spay/neuter) and age at first seizure were recorded in years. We defined a binary exposure variable indicating “gonadal removal by 5 years” with a temporality constraint to avoid reverse-causation in cases. Specifically, cases were classified as exposed only if gonadal removal occurred before seizure onset and before 5 years of age (Age gonadectomy < Age onset and Age gonadectomy < 5). Controls were classified as exposed if gonadal removal occurred before 5 years of age. Dogs not meeting the exposure definition (including intact dogs, dogs neutered at ≥5 years, and cases neutered after onset) were classified as unexposed. Odds ratios (ORs), 95% confidence intervals (CIs), and *p*-values were obtained from profile penalized likelihood inference, and overall model fit was summarized using a likelihood ratio test.

To evaluate age-specific risk of first seizure, we also fit a Cox proportional hazards model with age as the time scale. Time to event was age at first seizure for cases and age at last follow-up for controls (controls were right-censored). Gonadal removal before 5 years of age was implemented as a time-varying exposure using a counting-process (start–stop) data structure: dogs contributed a single interval from birth to the endpoint age if never neutered, neutered at ≥5 years, or neutered after seizure onset, and contributed two intervals if neutered before 5 years and prior to the endpoint—an unexposed interval from birth to age at neuter followed by an exposed interval from age neuter to the endpoint. For cases, the endpoint was age at seizure onset; for controls, it was age at last follow-up. Models were fit with age as the time scale and gonadectomy represented as a time-varying covariate (0 before gonadectomy, 1 after gonadectomy for dogs gonadectomized before 5 years of age and before the endpoint), adjusting for sex and clustering intervals by dog. Hazard ratios (HRs) with 95% CIs were reported, and proportional hazards assumptions were evaluated using Schoenfeld residuals (cox.zph). Sensitivity analyses were also performed using a stricter cutoff of 2 years of age to test whether the association was robust when gonadectomy was restricted to a narrow, prepubertal/peripubertal window and to evaluate whether results were being driven specifically by early procedures rather than by gonadectomy occurring anytime within the broader pre-onset period captured by the 5-year definition. All analyses were performed in R v4.1.2 using the logistf v1.26.1 [29] and survival v3.8.6 [31] packages.

### 2.5. Evolutionary Conservation and Population Frequency of the Lead Risk Marker

Top SNPs were annotated using the Ensembl Variant Effect Predictor, Ensembl release 115 [32]. To assess cross-species nucleotide conservation at the locus, the canine lead risk marker position (chr3:88,803,651; BICF2P1282800) was mapped to the corresponding orthologous coordinate in the human reference assembly (hg38) using the UCSC LiftOver tool (https://genome.ucsc.edu/cgi-bin/hgLiftOver, accessed on 1 February 2026). The mapped hg38 position was then interrogated in the UCSC Genome Browser using the Zoonomia 241-mammal whole-genome multiple Cactus alignment [33,34]. The aligned bases at the orthologous site were inspected across species to summarize nucleotide conservation within the order Carnivora.

Next, breed and subpopulation distribution were explored in an unascertained Arctic-lineage reference dataset from an unrelated previous study (355 canids including 279 Siberian Huskies) [19]. Genotype calls at the lead risk marker were extracted to calculate allele frequency (overall and within Siberian Huskies) and the frequency of homozygous risk allele carriers. Presence or absence of the identified risk-associated allele at the lead marker was also evaluated among sampled ancient Arctic dogs and wolves.

### 2.6. Local LD Structure and Haplotype/IBD Characterization

To further delineate the CFA3 association in the GWAS discovery cohort, regional LD patterns were summarized as LD to the lead risk marker (BICF2P1282800). Genotypes across CFA3:80–91 Mb were first extracted using PLINK2 [23,24]. LD was then computed in R as r^2^ between each regional SNP and the lead marker by taking the squared Pearson correlation of SNP dosage with lead-SNP dosage (calculated blockwise for efficiency), and these values were used to define LD bins for regional association plotting. Haplotype-based association testing was subsequently performed in R v4.1.2 using haplo.stats [35] across the three Bonferroni-significant SNPs (BICF2G630358500, BICF2P1282800, BICF2S23653217) spanning 88.3–88.9 Mb, estimating haplotype frequencies and testing association with idiopathic epilepsy using haplo.score, which provides a global test of haplotype association and haplotype-specific score statistics. Per-dog diplotypes and probabilities were inferred via the expectation–maximization (EM) algorithm. Because haplotype categories were sparse and quasi-complete separation was observed, haplotype effects were estimated using Firth-penalized logistic regression in logistf v1.26.1 [29]. Phased haplotypes were coded as dosages (0–2 copies) per haplotype, with haplotypes of frequency <0.05 collapsed; A-G-A was specified a priori as the reference and omitted to avoid collinearity. Models included PC1–PC2 from PLINK eigenvectors to control for population structure. Odds ratios and 95% confidence intervals were derived from exponentiated penalized coefficients and profile penalized log-likelihood limits.

Next, the CFA3 risk association was examined for evidence of a broader extended haplotype background. Although Extended haplotype homozygosity (EHH) and Unnormalized cross-population extended haplotype homozygosity (XP-EHH) are classically used to detect recent positive selection, the underlying quantities they measure are the extent and decay of haplotype homozygosity around a focal allele. We used these metrics here as complementary, descriptive tools to characterize the extent of shared haplotype structure across the associated CFA3 interval in risk and non-risk alleles. XP-EHH was computed at the lead marker using the R package rehh v3.2.2 [36] following the XP-EHH framework [37], contrasting risk-allele and non-risk-allele chromosomes within the Show/Pet group (where all but one carrier of the risk-associated marker was observed). SNP-array data were first filtered in PLINK2 to biallelic A/C/G/T SNPs only (excluding indels and non-ACGT allele codes) and a single shared SNP list for all of chromosome 3 was generated; these markers were exported as a VCF for the Show/Pet subset and phased with Beagle v5.4 [38] to obtain phased haplotypes without imputing untyped sites. The phased VCF was imported into rehh without ancestral allele polarization, and haplotypes were partitioned into risk and non-risk sets based on the allele carried at the lead-marker position. For each chromosome the median shared-tract length was calculated across all within-background pairwise comparisons (diagonal excluded). Distributions of these per-chromosome medians were then summarized separately for risk and non-risk allele backgrounds and visualized in R using ggplot2 v.4.0.1 [39]. Integrated haplotype homozygosity (iES) was computed within each set, and XP-EHH was calculated with ies2xpehh() using standardize = FALSE, yielding log(iES_Risk/iES_Non-Risk). Positive values indicate longer haplotypes (greater haplotype homozygosity) on risk-background chromosomes relative to non-risk chromosomes.

EHH decay curves were also computed in R using the rehh package v3.2.2 [36] to visualize how quickly haplotype homozygosity declines with distance from the CFA3 lead risk marker on risk versus non-risk chromosomes. These locus-centered curves complement XP-EHH and other haplotype/IBD analyses by directly displaying the local decay pattern around the risk-associated marker within a lineage. For each dataset, EHH was calculated (unpolarized; include_nhaplo = TRUE; discard_integration_at_border = TRUE) and summarized for the risk vs. non-risk backgrounds based on the allele (G vs. A) at BICF2P1282800 in the phased chromosomes. To assess sensitivity to unequal sample sizes, the Show/Pet control set was additionally analyzed after random down-sampling to 10 dogs (20 chromosomes) to approximate the number of risk-background chromosomes in this broad lineage (*n* = 19). EHH decay curves were visualized as EHH versus physical distance from the lead risk marker (Mb), with the lead position set to 0 Mb, in R using ggplot2 v4.0.1 [39].

Next, to evaluate whether the core CFA3 risk background showed evidence of recent shared ancestry consistent with spread of the associated haplotype from a recent common ancestor, identical-by-descent (IBD) segments were analyzed within the Show/Pet subset. Because all Bonferroni-significant SNPs localized within *KCNIP4*, IBD sharing was summarized across the more narrowly defined *KCNIP4*-centered association interval (chr3:87,771,875–88,894,884) to focus on the most strongly associated portion of the region, where shared IBD is more likely to reflect the risk haplotype rather than broader background ancestry. Hap-IBD [40] was run on phased haplotypes using a linear genetic map (1 cM/Mb) with minimum reported segment length of 0.5 cM and a minimum of 30 markers per segment. For each within-class haplotype pair (risk–risk or non-risk–non-risk), a pair was considered to share IBD across the *KCNIP4* interval (chr3:87,771,875–88,894,884) if at least one Hap-IBD segment overlapped the interval (segment end ≥ interval start and segment start ≤ interval end). Among sharing pairs, IBD sharing within the interval was summarized as the maximum genetic length (cM) of any overlapping Hap-IBD segment per haplotype pair, distributions of these per-pair maxima were summarized by the median, and the proportion of all possible within-class haplotype pairs with ≥1 segment overlapping *KCNIP4* was also reported.

Finally, genome-wide pairwise relatedness was estimated with the KING-robust kinship estimator in SNPRelate (snpgdsIBDKING) [41,42] using autosomal SNPs. Prior to kinship estimation, markers were LD-pruned using a correlation-based LD metric with an LD threshold of 0.2 across 500 kb sliding windows. Variants were additionally filtered to common, well-genotyped sites (MAF ≥ 0.05; missing rate ≤ 0.05). Kinship coefficients were computed from the LD-pruned autosomal marker set using SNPRelate’s default KING-robust settings to obtain a symmetric kinship matrix for all sample pairs. Carrier status for the CFA3 lead risk marker was defined using the dosage (≥1) of the A (risk) allele at BICF2P1282800. For each unique pair of dogs, the genome-wide KING kinship coefficient was extracted, and pairs were assigned to one of three categories: carrier–carrier, carrier–noncarrier, or noncarrier–noncarrier. To formally test whether carriers were more closely related to each other than noncarriers, the kinship distributions for carrier–carrier versus noncarrier–noncarrier pairs were compared using a two-sided Wilcoxon rank-sum test in R v4.2.1.

## 3. Results and Discussion

### 3.1. Genome-Wide Association Analysis and Dosage-Based Risk Modeling

**Figure 1:**
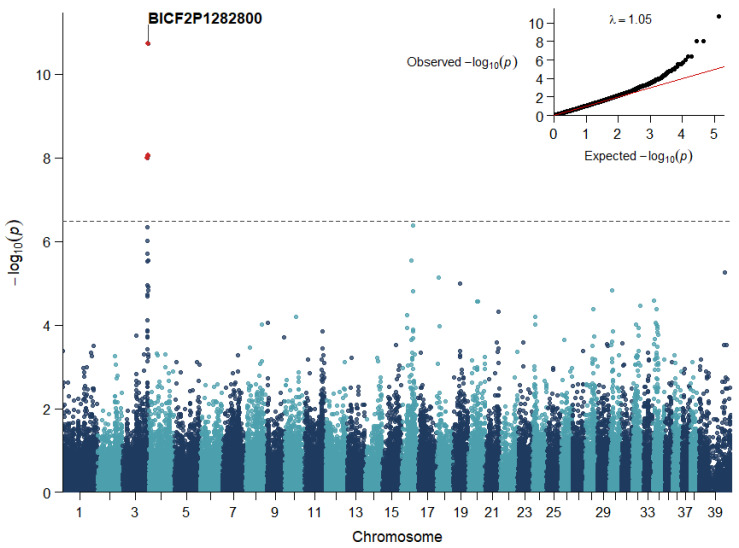
Genome-wide association results for idiopathic epilepsy in Siberian Huskies (*n* = 113). Association testing was performed in GEMMA [26] using a linear mixed model with a genomic relationship matrix to account for relatedness and PC1–PC2 included as fixed-effect covariates. Each point represents a genotyped SNP plotted by genomic position. The horizontal dashed line indicates the genome-wide Bonferroni threshold (*p* < 3.25 × 10^−7^). Inset: QQ plot showing deviation from the null distribution largely confined to the extreme tail.

**Figure 2:**
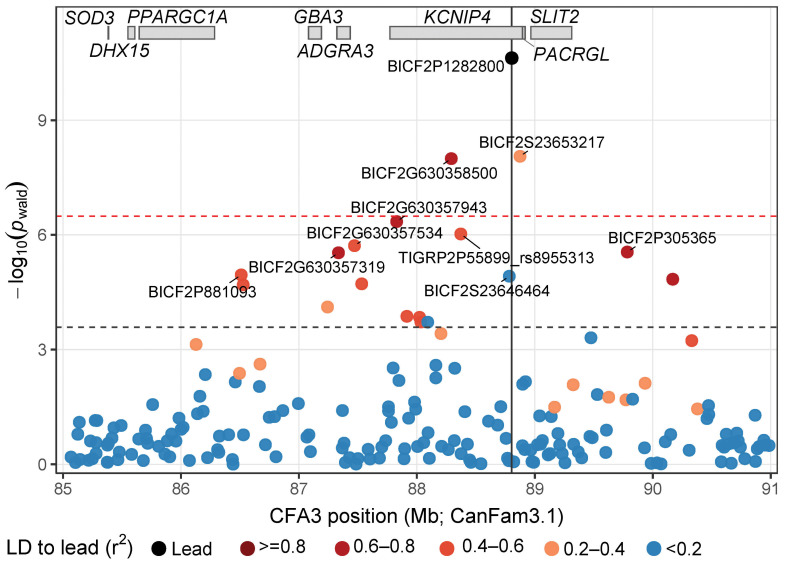
Zoomed regional view of the CFA3 GWAS signal shown in Figure 1. Points plot variant association strength from the GEMMA lmm across the implicated locus. The vertical line marks the lead risk marker highlighted in black, and point colors indicate linkage disequilibrium with BICF2P1282800 (r^2^). The red dashed horizontal line denotes the genome-wide Bonferroni threshold, and the black dashed line denotes the region-based threshold. Gene locations are shown as grey boxes above the plot to provide genomic context.

Mixed-model association testing identified a strong and localized association with idiopathic epilepsy on CFA3 centered within *KCNIP4* (Figure 1, Supplementary Table S3). The association signal comprised a cluster of correlated SNPs spanning ~2.5 Mb (87.3–89.8 Mb) with eight CFA3 markers exceeding a Benjamini–Hochberg False Discovery Rate (FDR) threshold of 0.05 (five within *KCNIP4*). A narrower core peak defined by three Bonferroni-significant SNPs spanned ~0.6 Mb (88.3–88.9 Mb; Figure 2), all within *KCNIP4*. At the lead risk marker, BICF2P1282800 (chr3:88,803,651; CanFam3.1; G > A), the GEMMA lmm estimated an additive effect of β = 0.531 (SE = 0.07; Wald *p* = 1.9 × 10^−11^), and nearby SNPs in the same interval within *KCNIP4* showed similarly strong support (Supplementary Table S3, Figure 2). Including PC1–PC2 as fixed-effect covariates in the GEMMA lmm reduced genomic inflation (λGC 1.11→1.05) without attenuating the CFA3 peak, and Quantile–quantile (QQ) plots showed deviation confined to the extreme tail, consistent with a true association signal (Figure 1). Additionally, no CFA37 association peak was observed, providing no evidence for the common *ADAM23* risk variant in this breed.

Because purebred dogs may show substantial relatedness and strong within-breed population structure, the CFA3 association was examined to assess whether it could be attributable to residual lineage stratification. However, we found that the signal remained stable across multiple sensitivity analyses. In the mixed-model framework, expanding covariate adjustment from PC1–PC2 to PC1–PC5 did not materially alter the CFA3 peak, indicating robustness to more stringent control of subtle population structure. Sensitivity to subpopulation structure was further assessed using a Cochran–Mantel–Haenszel (CMH) test in PLINK v1.9 [23,24], which estimates association while stratifying by owner-reported lineage (pet, show, racing, and Seppala)—subpopulations previously shown to be genetically differentiated in this breed [19]. The lead marker (BICF2P1282800) remained significantly associated after stratification (CMH χ^2^ = 26.5, *p* = 2.71 × 10^−7^; OR = 15.8), and a Breslow–Day test provided no strong evidence of effect heterogeneity across strata (*p* = 0.129). In a conditional mixed-model analysis that included lead-marker dosage as a fixed-effect covariate, the regional CFA3 association was eliminated, consistent with a single primary signal tagged by the lead marker rather than multiple independent effects. Likewise, the zoomed regional view in Figure 2 showed that the other CFA3 significant SNPs were in moderate to strong LD with the lead marker, supporting the interpretation that the CFA3 association reflects a single associated interval. Finally, restricting the mixed-model analysis to the Show/Pet subset alone (*n* = 58; 17 cases) as a sensitivity analysis yielded a similarly significant and localized CFA3 peak centered at 88.3–88.9 Mb within *KCNIP4* (minimum Wald *p* = 4.16 × 10^−8^; Supplementary Table S4); this within-lineage signal was likewise stable to inclusion of additional PCs (PC1–PC5). The persistence of the same localized CFA3 signal across the full cohort and the Show/Pet subset, together with concordant results from multiple structure-control analyses, argue against population structure as the primary driver of the CFA3 association and support BICF2P1282800 as a robust risk-associated marker tagging the CFA3 interval centered within *KCNIP4*. Although a false-positive association cannot be fully excluded in the absence of replication in an independent cohort, the consistency of the signal across these complementary analyses makes it unlikely that the observed association is explained solely by residual lineage stratification.

Next, case–control genotypes at the lead risk marker were compared to measure how enriched the risk allele was in affected dogs and to test whether risk increased with allele dosage. Genotype counts at BICF2P1282800 (Table 1) showed pronounced enrichment of the lead marker risk allele (A) among IE cases in the discovery cohort (*n* = 113): 13/24 cases (54.2%) carried at least one A allele compared with 3/89 seizure-free controls (3.4%), and no controls were homozygous AA. Genotype counts also supported a clear allele-dosage pattern in this pooled (GWAS + Sanger) dataset (*n* = 170): among heterozygotes (dosage 1), 19/31 (61%) were affected, and all (5/5) homozygous-risk dogs (dosage 2) were affected, consistent with increasing penetrance with additional risk alleles (Table 1). Because no seizure-free controls carried the risk-homozygous genotype (dosage = 2), we fit Firth-penalized logistic regression models including cohort (GWAS vs. Sanger) as a covariate. This yielded a strong additive dosage association in the pooled dataset (β = 1.88, SE = 0.39. OR = 6.6, *p* = 7.1 × 10^−8^). Standard logistic regression produced concordant inference (β = 1.95, SE = 0.40; OR = 7.0, *p* = 1.1 × 10^−6^), reinforcing a major, dosage-sensitive risk marker on CFA3 centered within the *KCNIP4* locus in Siberian Huskies. In plain terms, each additional risk allele was associated with ~7-fold higher odds of epilepsy in this cohort, and the association was highly unlikely to be due to chance.

In the pooled dataset, carriage of the lead risk marker was uncommon among seizure-free older controls (>7.5 years; 12/122, 9.8%), and all control carriers were heterozygous (Table 1). Importantly, the Sanger follow-up cohort was intentionally ascertained to enrich for affected dogs and their close relatives to evaluate within-family segregation and variable clinical expression; therefore, marker frequencies in that cohort should be interpreted in the context of this non-random ascertainment. In addition, a substantial proportion of affected dogs did not carry the lead risk marker (24/48, 50%). These observations are consistent with incomplete penetrance of marker-defined genetic risk and/or etiologic heterogeneity (e.g., additional risk loci), and it may also reflect imperfect tagging of the causal variant by the lead marker if recombination has reduced linkage between the marker and the causal mutation. Incomplete penetrance may further reflect non-genetic or gene–environment modifiers, including sex-related hormonal effects, age at gonadectomy, and other management or physiologic factors that may influence seizure threshold in genetically susceptible dogs. Thus, in this study, the lead marker identifies a substantial subset of risk (present in half of affected dogs) while also implying additional genetic contributors and biologic/environmental modifiers of disease expression.

**Figure 3:**
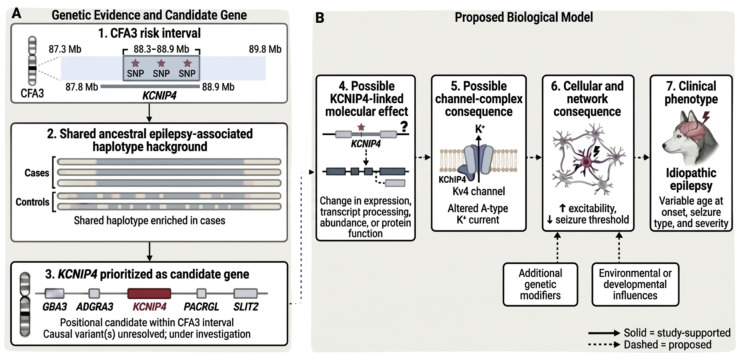
Genetic evidence and proposed biological model for the CFA3 epilepsy-associated interval in Siberian Huskies. (**A**) The CFA3: 87.3–89.8 Mb interval is associated with idiopathic epilepsy and contains a shared epilepsy-associated haplotype background. Within this broader associated region, the Bonferroni-significant signal localizes to CFA3:88.3–88.9 Mb. *KCNIP4* is shown as a positional candidate gene within the interval. (**B**) The associated SNPs are proposed to tag linked causal variant(s) that may affect *KCNIP4* regulation or function. These effects could alter neuronal excitability, lower seizure threshold, and contribute to hyperexcitable networks that promote seizure initiation and propagation, resulting in the idiopathic epilepsy phenotype. Solid arrows denote study-supported relationships; dashed arrows denote hypothesized downstream effects. This image was created in BioRender (https://BioRender.com, accessed on 6 April 2026).

*KCNIP4* is the strongest candidate gene within the associated CFA3 interval based on both positional evidence and biological plausibility, given that it encodes a neuronal voltage-gated potassium (Kv) channel–interacting protein (KChIP4) that regulates Kv-channel function and modulates membrane excitability [43,44]. Broadly, pathogenic variation in Kv channels and their auxiliary interacting proteins have been implicated in seizure disorders across multiple species [43,44,45,46,47,48,49]. For example, *Kcnip2* knockout mice show increased hippocampal excitability and greater seizure susceptibility [47], and altered expression of KChIP1, KChIP2, and KChIP3 has been reported in murine epilepsy models and human epileptic tissue [48,49]. In dogs, although the clinical presentation differs from epilepsy, *KCNIP4* has been linked to a breed-specific inherited neurologic disorder in Norwegian Buhunds, where a missense variant segregates with cerebellar ataxia [50]. Consistent with a role in seizure-relevant circuitry, single-nucleus RNA-seq data from the canine hippocampus demonstrate that *KCNIP4* is highly and specifically expressed in glutamatergic and GABAergic neurons in the canine brain (Supplementary Figure S1) [51,52]. Together, these lines of evidence support *KCNIP4* as a mechanistically plausible epileptogenic candidate and provide the basis for the plausible proposed biological model shown in Figure 3, making this gene a compelling target for causal variant discovery and functional validation. However, *SLIT2*, located immediately downstream of *KCNIP4*, warrants consideration as a secondary candidate given its proximity to the association signal and its established roles in axon guidance, neuronal migration, and neural circuit formation [53].

Several additional loci approached genome-wide significance (Supplementary Table S3), including a secondary peak on CFA16 near *MICU3* that came closest to the Bonferroni threshold. The lead CFA16 marker at BICF2S23437988 maps within *MICU3* and showed a strong effect estimate (β = 0.655; Wald *p* = 4.08 × 10^−7^), with an adjacent CFA16 signal providing supportive local concordance. Because *MICU3* regulates mitochondrial Ca^2+^ entry in neurons to support activity-dependent ATP production and metabolic flexibility [54], variation affecting *MICU3* function could plausibly influence neuronal energetics during high activity states, a mechanistic context relevant to hyperexcitability and seizure susceptibility [55]. While this CFA16 association did not surpass the genome-wide multiple-testing threshold in this cohort and should therefore be interpreted cautiously, it represents a possible secondary candidate locus for replication in independent cohorts and for follow-up fine-mapping/sequence-based interrogation to evaluate if it reflects an additional risk factor or possibly lineage-specific heterogeneity.

### 3.2. Age-at-Onset Distribution and Modifier Analyses in CFA3 Risk-Allele Carriers

**Figure 4:**
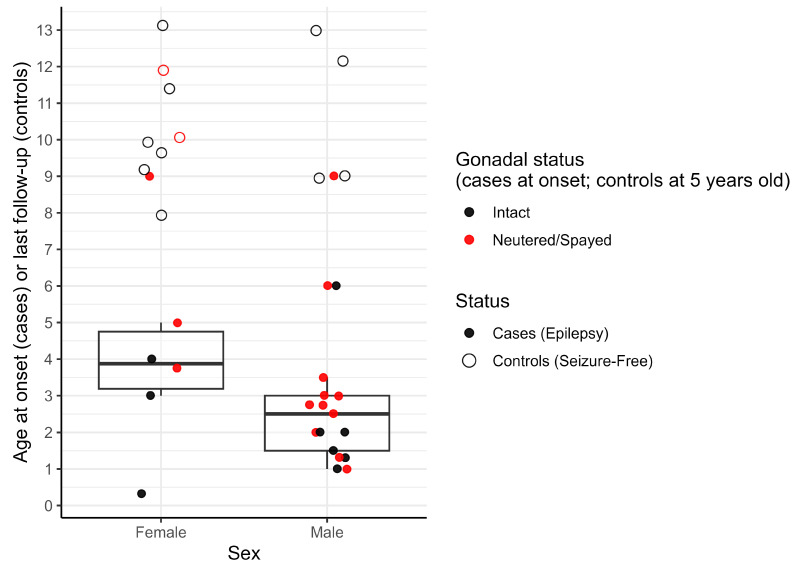
Distribution of age at seizure onset (cases) and age at last follow-up (seizure-free controls) among risk-allele carriers by sex and gonadal status (*n* = 35). Boxplots summarize age at seizure onset for cases only, stratified by sex and colored by gonadal status at seizure onset (intact vs. neutered/spayed). The center line indicates the median, boxes indicate the interquartile range (IQR), and whiskers extend to 1.5 × IQR. Open circles indicate seizure-free heterozygous carrier controls (dose = 1) overlayed onto plot at age at last follow-up, positioned by sex and colored by gonadal status at age 5 years.

Given the observed incomplete penetrance among carriers, we next evaluated sex and gonadal status as potential modifiers by examining age at seizure onset in relation to sex and age/timing of gonadectomy (neuter/spay). Among risk allele carriers (dosage ≥ 1) with recorded age that developed epilepsy (*n* = 23), age at seizure onset clustered in early adulthood (Figure 4). Females (*n* = 6) had a later median age at seizure onset than males (*n* = 17) (median 3.88 vs. 2.50 years), and the interquartile range (IQR) was different between sexes (females: 3.2–4.8 years; males: 1.5–3.0 years), indicating that the central 50% of male onsets occurred earlier.

Among *KCNIP4* risk-allele carriers (dosage ≥ 1) with gonadectomy information (*n* = 35), gonadectomy before 5 years of age (and before seizure onset in cases) was observed more often in cases than in controls (14/23 vs. 2/12; Figure 4). In Firth penalized logistic regression restricted to carriers, with epilepsy case status as the outcome and gonadectomy timing plus sex as predictors, overall model fit was significant (likelihood ratio test *p* = 0.0065; Supplementary Table S5). Under the <5-year definition, gonadectomy before seizure onset was associated with higher odds of epilepsy case status (OR 5.98, 95% CI 1.25–40.02, *p* = 0.024), and male sex was also associated with higher odds (OR 4.77, 95% CI 1.05–25.76, *p* = 0.043). Under the stricter <2-year definition, the association with gonadectomy was modestly attenuated, although this likely also reflects reduced power due to the smaller number of exposed dogs.

We then fit Cox proportional hazards models using age as the time scale and modeled gonadectomy as a time-varying exposure to evaluate age-specific risk of first seizure, adjusting for sex with robust standard errors clustered by dog (Supplementary Table S5). In the <5-year model, gonadectomy before 5 years of age was associated with increased hazard of first seizure (HR 2.69, 95% CI 1.08–6.73, *p* = 0.034), and male sex was also associated with increased hazard (HR 3.12, 95% CI 1.33–7.32, *p* = 0.0089). Under the stricter <2-year definition, the estimated gonadectomy effect remained in the same direction but was weaker (HR 2.35, 95% CI 0.97–5.71, *p* = 0.0595), whereas the male effect remained stable. Collectively, these analyses support an association in this sampled carrier cohort between male sex and epilepsy-related outcomes, with more tentative evidence that gonadectomy before 5 years of age may be associated with increased epilepsy odds and earlier seizure onset. Although the observed pattern is compatible with hormonal modulation of seizure susceptibility, as sex hormones and their neurosteroid metabolites can influence neuronal excitability and seizure threshold [30,56,57,58], these findings should be viewed as hypothesis-generating rather than causal. This interpretation should remain cautious because the analysis is observational, the sample size is modest, controls were enriched for older seizure-free carriers, and residual confounding cannot be excluded. In particular, reverse causation remains possible, as subtle neurologic signs, pre-diagnostic behavioral changes, or owner concern regarding familial seizure risk may have influenced retention in breeding programs or the timing/decision to gonadectomize. Selection bias may also have contributed, because dogs that were neutered/spayed before 5 may have differed systematically from dogs that remained intact in ways related to veterinary care and/or owner monitoring, which could influence epilepsy detection and classification independently of any biological effect of gonadectomy. In addition, clinical decision-making influences cannot be excluded, as recommendations regarding gonadectomy may have been shaped by emerging behavioral or health concerns prior to seizure onset. Replication in larger, independently ascertained carrier cohorts with standardized longitudinal follow-up will be needed to refine effect sizes and assess robustness.

### 3.3. Evolutionary Conservation and Population Frequency of the Lead Risk Marker

Next, to place the lead association in a functional and evolutionary context, the genomic position and evolutionary conservation of BICF2P1282800 was evaluated. Annotation with the Ensembl Variant Effect Predictor (VEP) [32] localized the lead marker deep within intron 2 of *KCNIP4* in the canonical transcript. A comparative genomic analysis indicates that this intronic position lies within a region of strong evolutionary constraint: in the Zoonomia 241-mammal alignment, 29 of 30 carnivore genomes, including all examined canids, retain a conserved guanine at the orthologous site, consistent with long-term purifying selection acting on this locus and/or its surrounding sequence context [33,34].

To contextualize the lead marker risk allele outside an epilepsy-enriched cohort, its background frequency was examined in an unascertained Arctic-lineage reference dataset comprising 355 canids (including 279 Siberian Huskies) from Smith et al. [19]. In this panel, the allele was uncommon (overall allele frequency = 0.075; 0.068 in Siberian Huskies; Table 2), was not observed among the sampled ancient Arctic dogs or wolves, and homozygosity was rare (one Siberian Husky). Notably, the sole homozygous individual was owner-reported to have idiopathic epilepsy, providing external concordance with the association detected in the present study. Collectively, the low population frequency and the scarcity of homozygotes in an unselected Arctic-lineage dataset is consistent with the interpretation that the marked case enrichment reflects a genuine disease-associated risk background in this breed.

Beyond establishing that the risk allele at the lead risk marker is relatively rare in Arctic-lineage dogs, the reference dataset also provides a framework to evaluate whether its occurrence is uniform within the breed or concentrated within certain subpopulations. This lineage pattern can help evaluate whether the allele is consistent with an older, broadly distributed variant present before or early in breed divergence—or instead shows clear enrichment in particular subpopulations, consistent with a more recent origin or, alternatively, recent demographic amplification. To evaluate this, the risk allele frequency at the lead risk-associated marker was assessed within owner-reported subpopulations in the Siberian Husky breed in both this study and the Arctic-lineage reference dataset. Table 2 shows marked subpopulation differences in the frequency of the risk-associated marker allele in both. The allele is enriched in mixed/pet/show lineages and rare to absent in working-only subpopulations (racing/Seppala) in both cohorts. Although absolute frequencies are expected to differ between datasets due to intentional case enrichment in the present study, both datasets show the same overall pattern. This motivated haplotype-based analyses to test for evidence of a recent expansion.

### 3.4. Local LD Structure and Haplotype/IBD Characterization

Given the marked subpopulation differences in lead-marker risk allele frequency, haplotype analysis was not only used to refine the shared candidate interval for future fine-mapping, but also to test whether the CFA3 signal represents recent expansion of a linked de novo mutation that arose more recently in the Pet/Show group, versus an older risk allele present on multiple haplotype backgrounds and differentially amplified across lineages. If the signal reflects recent expansion of a newly arisen mutation, carriers are expected to be more closely related than noncarriers, and to share a long, relatively homogeneous haplotype/IBD tract spanning the locus, with limited evidence of multiple distinct surrounding haplotype backgrounds. In contrast, if the linked causal mutation is substantially older (e.g., predating formal breed formation around 1930) but was only recently amplified in some subpopulations through demographic processes, recombination over many generations would be expected to have broken down the linkage such that the causal mutation occurs on multiple haplotype backgrounds. This would yield greater heterogeneity among haplotypes and overall relatedness among carriers, including a mix of shorter and longer shared tracts around the locus, and potentially lineage-specific risk haplotypes in which the lead marker may only imperfectly track the causal variant.

To test this, and to evaluate whether the CFA3 signal is better represented by a multi-marker background than by a single lead SNP, phased haplotypes were first inferred across the three Bonferroni-significant SNPs spanning the ~0.6 Mb association peak (Supplementary Table S1) in the discovery cohort using the R package haplo.stats [35]. Haplotype distributions differed strongly between cases and controls (haplo.stats global score test *p* = 6.78 × 10^−9^), driven primarily by the T-A-G haplotype (frequency 0.08), which was markedly enriched in cases (score = 6.20; *p* = 5.80 × 10^−10^). In contrast, the common A-G-A haplotype (frequency 0.68) was enriched in controls (score = −4.31; *p* = 1.60 × 10^−5^), consistent with depletion among cases in the presence of a case-enriched risk background (Supplementary Table S1). Using A-G-A as the reference haplotype, a PC-adjusted Firth-penalized haplotype-dosage model identified T-A-G as the primary case-enriched haplotype, with a large increase in IE odds per copy (OR = 26.9, *p* = 1.97 × 10^−8^). Because these haplotypes were statistically inferred rather than directly observed, some assignments carried posterior probabilities <1 (Supplementary Table S1), indicating residual phase uncertainty; however, this uncertainty is expected mainly to reduce the precision of downstream haplotype-dosage estimates rather than alter the overall interpretation. Practically, this indicates that the CFA3 signal is well captured by a short core haplotype, and that either the lead SNP or the T-A-G haplotype can serve as a proxy for the underlying risk variant in this cohort.

**Figure 5:**
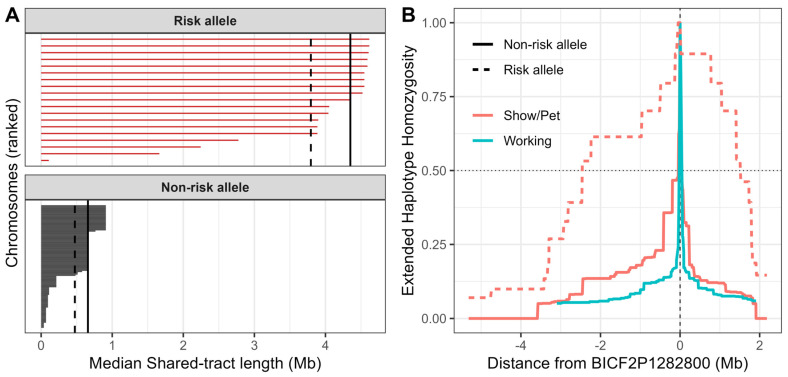
Extended haplotype homozygosity across the CFA3 region in Siberian Husky lineages. (**A**) Phased chromosomes were partitioned by the allele carried at the lead risk marker (CFA3:88,803,651; BICF2P1282800) into risk allele (**top**; red) and non-risk allele (**bottom**; gray) backgrounds in the Show/Pet subset. Each horizontal segment represents a single phased chromosome (haplotype), ranked by length within panel, and the x-axis shows the median shared haplotype-tract length (Mb) with all other chromosomes carrying the same allele, computed from pairwise shared-tract lengths extending on both sides of the lead marker. Vertical reference lines denote the panel median (solid) and panel mean (dashed) shared-tract length. Longer shared tracts among risk chromosomes indicate increased haplotype sharing consistent with a common founder-derived risk haplotype. (**B**) EHH is plotted as a function of physical distance (Mb) from BICF2P1282800 (vertical dashed line at 0). Curves are shown separately for Show/Pet (red) and Working (teal) subsets, and for haplotypes carrying the non-risk allele (solid lines) versus the risk allele (dashed lines). The horizontal dotted line marks EHH = 0.5.

Direct quantification of local haplotype sharing around the lead risk marker further demonstrated risk-associated chromosomes shared markedly longer tracts than non-risk chromosomes (median per-chromosome shared-tract length 4.35 Mb vs. 0.66 Mb; Figure 5A). Likewise, extended haplotype homozygosity (EHH) analyses showed a broad region of positive values spanning ~86–90 Mb (Supplementary Figure S2), consistent with increased haplotype homozygosity and extended haplotype sharing across a multi-megabase interval overlapping the lead marker on the risk background. Outside this interval, XP-EHH values across the remainder of CFA3 did not show comparably elevated signals, indicating that the extended haplotype pattern is localized rather than a chromosome-wide artifact. EHH decay was also markedly decreased on risk chromosomes, falling below 0.5 at ~86.34 Mb (2.46 Mb upstream) and ~90.32 Mb (1.52 Mb downstream), corresponding to a half-decay span of ~3.98 Mb (Figure 5B). In contrast, EHH on non-risk chromosomes dropped below 0.5 within a narrow interval in both Show/Pet (~0.090 Mb) and Working non-risk controls (~0.070 Mb). This contrast persisted after down-sampling to equalize haplotype counts (Supplementary Figure S3), arguing against unequal sample size as the cause and instead indicating that the risk allele is carried on a much longer, more uniformly shared haplotype than the non-risk background. Together, these results suggest the CFA3 association reflects a long, shared risk haplotype carried by many cases, consistent with recent shared ancestry, and that there has been insufficient time (or limited effective recombination) to break the region into smaller segments.

To test the hypothesis of recent shared ancestry, Hap-IBD [40] was used, a haplotype-based identity-by-descent method that detects long genomic segments shared between individuals due to inheritance from a recent common ancestor. Hap-IBD provided direct evidence of recent shared ancestry among risk chromosomes: 117 of 171 risk–risk haplotype pairs (68.4%) shared an IBD segment overlapping *KCNIP4*, with a median maximum overlap of 4.46 cM, supporting inheritance of a common risk-associated chromosome segment in many carriers. Using shared IBD tract length as a rough guide to how recently carrier chromosomes share a common ancestor, the median shared segment across the risk region (4.46 cM) is consistent with a common ancestor on the order of ~11 generations ago (using the heuristic g ≈ 50/L_cM_). Importantly, these tract-based estimates date the recent expansion/common ancestry of the shared risk haplotype background, and do not necessarily indicate when the causal mutation first arose (which could predate the recent rise in frequency). These are approximate time scales as local variation in recombination rate and IBD-calling thresholds can shift the estimates, but they support the same overall conclusion of recent shared ancestry.

We next evaluated whether the observed IBD sharing could be explained by carriers simply being closely related overall. To test this, genome-wide KING kinship estimates were used to compare relatedness among dogs carrying the lead risk marker versus dogs lacking it, which helps distinguish a broadly distributed risk haplotype from a signal driven primarily by a few closely related families. Risk carrier–carrier pairs spanned a wide range of genome-wide KING kinship coefficients (−0.53 to 0.15; Supplementary Figure S4). Despite this heterogeneity, carrier–carrier pairs were modestly more related to one another than noncarrier–noncarrier pairs (median −0.0995 vs. −0.133), and the difference was significant (Wilcoxon rank-sum: W = 317,282; *p* = 0.011). The presence of some positive kinship values among carrier–carrier pairs (up to 0.15, consistent with approximately second-degree relatedness) indicates that a subset of carriers are closely related, as expected in a closed breeding population. However, the kinship distribution is centered near zero and largely negative, with many pairs showing strongly negative values. This indicates that most carrier–carrier pairs are not close relatives, consistent instead with a more distant shared ancestor on the order of ~11 generations ago, and with a risk allele that is distributed more broadly within the population, rather than being confined to a small number of close relatives.

Collectively, the haplotype, IBD, and kinship analyses indicate that the CFA3 signal is capturing a long, shared risk haplotype that is carried by many dogs with IE within the breed. The multi-megabase XP-EHH peak and extended half-decay span around the lead marker, together with frequent IBD sharing across *KCNIP4* and slightly higher genome-wide relatedness among carriers than noncarriers, indicate that risk-allele chromosomes remain unusually similar across several megabases. In the context of disease, these findings are consistent with the associated CFA3 interval tagging a shared haplotype that contributes to epilepsy risk, with a predominant epilepsy-associated chromosome background appearing to have expanded primarily in mixed/pet/show lineage dogs. At the same time, the presence of a minority of lead marker-positive chromosomes carrying different haplotypes (Supplementary Table S1), with substantially shorter homozygous tract lengths (Figure 5A), is consistent with an older origin of the tagged causal mutation that has been carried on multiple local backgrounds and recently amplified. Limited gene flow among subpopulations, combined with breeding practices that concentrate ancestry (e.g., popular-sire use) and drift in smaller or bottlenecked pools, could rapidly increase the frequency of a rare risk haplotype in one lineage while it remains uncommon in another. Stronger selection against neurologic disease in working dogs could also contribute, but because idiopathic epilepsy still occurs in working-line dogs (Supplementary Tables S1 and S2), selection alone is unlikely to fully explain the pronounced lineage differences.

This lineage-specific amplification model also provides a framework for interpreting affected dogs that do not carry the lead risk marker. Dogs that do not descend from the recently expanded risk-haplotype background, such as many working-line dogs that may have diverged from show/pet lineage dogs earlier than 11 generations ago, would not be expected to carry the same long shared chromosome segment captured by multiple linked markers on the CanineHD array. Notably, however, the single risk-allele chromosome observed in the Working group does not appear more recombined (median shared tract length = 4.62 Mb) than Show/Pet risk chromosomes (Supplementary Figure S5), suggesting it likely reflects recent introgression of the same risk-haplotype background rather than an independent, long-diverged working-line risk chromosome; consistent with this, pedigree inspection identifies multiple show champion ancestors approximately nine generations back in this dog’s pedigree. If the causal mutation is older but rose to higher frequency only recently through demographic expansion in the Show/Pet group, the lead marker may tag risk well within the recently amplified haplotype background but less well outside it. Consequently, the marker’s predictive value is expected to be lineage dependent—high in subpopulations carrying the amplified risk chromosome, or in pedigrees with introgression from that background, but reduced in lineages that do not share that recently amplified ancestral segment. Importantly, however, because extended haplotypes can also arise from recombination-suppressing structural variation (e.g., inversions), high-coverage sequencing will be required to disentangle these mechanisms and better resolve the evolutionary history of the locus.

## 4. Conclusions

This study prioritizes a CFA3 interval centered within *KCNIP4* as a major risk locus for idiopathic epilepsy in Siberian Huskies, with the lead risk-associated marker present in approximately half of cases and showing a pronounced allele–dose relationship. Convergent evidence from linear mixed-model association, lineage-stratified sensitivity analyses, and haplotype/IBD-based evaluations all point to the same conclusion: many affected dogs carry the same long risk haplotype, likely inherited from a recent common ancestor(s). This risk haplotype is much more common in mixed/pet/show lines than in working lines, and the data are consistent with it having increased in frequency relatively recently in those subpopulations. Given incomplete penetrance among heterozygotes and the presence of affected non-carriers, the locus is best interpreted as a strong, but not exclusive, contributor to disease liability, implying recombination between the risk marker and causative mutation, or additional genetic and/or non-genetic determinants within the breed. However, an important limitation is that the association has not yet been replicated in an independent cohort, which limits confidence in the broader generalizability of the findings beyond the sampled population. Future work should prioritize independent replication and high-coverage sequencing across the associated interval to resolve risk-haplotype structure and identify candidate causal variants, followed by functional validation of prioritized mechanisms to establish molecular consequence and causality. At present, these findings do not support commercial use of the lead risk marker as a predictive test. Until the underlying causative variant(s) are identified and functionally validated, application of this marker in commercial testing would be premature.

## Figures and Tables

**Table 1:** Distribution of risk-allele dosage at the lead risk marker, BICF2P1282800, in the GWAS, Sanger and Pooled cohorts. Counts show the number of idiopathic epilepsy cases and seizure-free controls carrying 0, 1, or 2 copies of the risk allele.

Lead Risk Marker BICF2P1282800 Dosage	GWAS Cohort *n* = 113		Sanger Cohort *n* = 57		Pooled Dataset *n* = 170	
	Cases (*n* = 24)	Controls (*n* = 89)	Cases (*n* = 24)	Controls (*n* = 33)	Cases (*n* = 48)	Controls (*n* = 122)
0	11	86	13	24	24	110
1	9	3	10	9	19	12
2	4	0	1	0	5	0

**Table 2:** Risk-associated marker allele frequency by Siberian Husky subpopulation across cohorts. Shown are the frequencies of the CFA3 lead-marker risk allele within owner-reported Siberian Husky subpopulations in this study and in the external unascertained Arctic-lineage dataset reported by Smith et al. [19]. Values are given as percentage risk-allele frequency with the number of risk alleles observed over the total number of chromosomes assessed in parentheses. “Show/Sled-Show,” “Pet,” “Racing,” and “Seppala” denote owner-reported lineages; “Husky Mix” includes non-registered mixed-breed dogs with ≥50% Siberian Husky ancestry.

Siberian Husky Subpopulation	This Study	Smith et al., 2024 [19]
Show/Sled-Show	14.4% (21/146)	9.4% (23/246)
Pet	22.7% (15/66)	14.3% (6/42)
Racing	0.9% (1/106)	4.1% (9/222)
Seppala	0% (0/16)	0% (0/48)
Husky Mix	66.7% (4/6)	NA
**Total**		**6.8% (38/558)**

## Data Availability

De-identified data presented in this study are available on request from the corresponding author to academic researchers only due to confidentiality considerations.

## Supplementary Materials

The following supporting information can be downloaded at: https://www.mdpi.com/article/10.3390/genes17040459/s1, Supplementary Table S1. Individual-level haplotype assignments for the 113 Siberian Huskies included in the GWAS [35]. Supplementary Table S2: Individual-level genotype assignments for the 73 Siberian Huskies that were Sanger sequenced. Supplementary Table S3. SNPs associated with idiopathic epilepsy in Siberian Huskies after Benjamini–Hochberg false discovery rate correction. Supplementary Table S4: Genome-wide association results for idiopathic epilepsy in the Show/Pet subset of Siberian Huskies. Supplementary Table S5: Association of gonadal removal with epilepsy status and age-at-onset among CFA3 risk-allele carriers under two definitions (<5 years vs. <2 years). Supplementary Figure S1: Single-nucleus expression of *KCNIP4* in the canine hippocampus [51,52]. Supplementary Figure S2: Unnormalized XP-EHH across CFA3 in the Show/Pet group. Supplementary Figure S3: Extended haplotype homozygosity (EHH) decay centered on the lead risk marker. Supplementary Figure S4: Genome-wide relatedness by CFA3 risk-marker carrier status. Supplementary Figure S5: Median shared-tract length among risk chromosomes by subpopulation.

## Abbreviations

The following abbreviations are used in this manuscript:

CFA	*Canis familiaris* autosome
GWAS	Genome Wide Association Study
EHH	Extended haplotype homozygosity
IBD	Identical by descent
IE	Idiopathic epilepsy
LD	Linkage disequilibrium
SNP	Single nucleotide polymorphism
XP-EHH	Cross-population extended haplotype homozygosity
