# Supplementary material for: A Novel CFA3 Locus Encompassing KCNIP4 Is Associated with Idiopathic Epilepsy in Siberian Huskies
Source: Genes (Basel). 2026 Apr 15;17(4):459. doi: 10.3390/genes17040459 (PMC13115692; doi:10.3390/genes17040459)
Supplement: Supplementary file 1 [file genes-17-00459-s001.zip › genes-4237803-supplementary.pdf]

Supplementary Table S1. Individual-level haplotype assignments for the 113 Siberian Huskies included in the GWAS, based on the three Bonferroni-significant CFA3 SNPs (BICF2G630358500, BICF2P1282800, BICF2S23653217). For each dog, the table reports population, subject ID, case/control status, the inferred phased diplotype (Hap 1 and Hap 2) across these three markers, and the posterior probability of that assignment. Haplotype phase was estimated using the EM algorithm implemented in haplo.stats (Sinnwell & Shaid, 2024), and the diplotype with the highest posterior probability is shown for each individual.

Population	Subject ID	Status	Hap 1	Hap 2	Posterior_prob
HuskyMix	G1	case	T-A-G	T-A-G	1
HuskyMix	G2	case	T-A-G	T-A-G	1
Pet	G3	control	A-G-A	A-A-G	1
Pet	G4	case	T-A-G	A-G-G	0.997662
Pet	G5	case	A-G-A	A-G-A	1
Pet	G6	control	A-G-A	T-A-G	0.997034
Pet	G7	control	A-G-A	A-G-A	1
Pet	G8	control	A-G-A	A-G-A	1
Pet	G9	control	A-G-A	A-G-A	1
Pet	G10	case	T-A-G	A-G-G	0.997662
Pet	G11	control	A-G-A	A-G-A	1
Pet	G12	control	A-G-A	A-G-A	1
Pet	G13	control	A-G-A	A-G-A	1
Pet	G14	control	A-G-A	A-G-A	1
Pet	G15	case	T-A-G	A-G-A	0.997034
Pet	G16	case	A-G-A	A-A-G	1
Pet	G17	case	A-G-A	A-G-A	1
Pet	G18	control	A-G-G	A-G-A	1
Pet	G19	control	A-G-A	A-G-A	1
Pet	G20	control	A-G-A	A-G-A	1
Pet	G21	case	T-A-G	A-G-A	0.997034
Pet	G22	control	A-G-A	A-G-A	1
Racing	G23	control	A-G-A	A-G-G	1
Racing	G24	control	A-G-A	A-G-G	1

Racing	G25	control	A-G-G	A-G-A	1
Racing	G26	control	A-G-G	A-G-A	1
Racing	G27	control	A-G-A	A-G-A	1
Racing	G28	control	A-G-A	A-G-G	1
Racing	G29	control	A-G-A	A-G-G	1
Racing	G30	control	A-G-G	A-G-A	1
Racing	G31	control	A-G-A	A-G-G	1
Racing	G32	control	A-G-G	A-G-A	1
Racing	G33	control	A-G-G	A-G-G	1
Racing	G34	case	A-G-G	A-G-G	1
Racing	G35	control	A-G-G	A-G-G	1
Racing	G36	control	A-G-A	A-G-A	1
Racing	G37	control	T-G-A	A-G-A	1
Racing	G38	control	A-G-A	A-G-A	1
Racing	G39	control	A-G-G	A-G-G	1
Racing	G40	control	T-G-A	A-G-A	1
Racing	G41	case	A-G-G	A-G-G	1
Racing	G42	control	A-G-A	A-G-A	1
Racing	G43	control	A-G-G	A-G-G	1
Racing	G44	case	A-G-G	A-G-G	1
Racing	G45	control	A-G-A	T-G-A	1
Racing	G46	control	A-G-G	A-G-G	1
Racing	G47	control	T-G-G	A-G-G	1
Racing	G48	control	A-G-G	A-G-A	1
Racing	G49	control	A-G-A	A-G-A	1
Racing	G50	control	A-G-G	A-G-A	1
Racing	G51	control	A-G-A	A-G-G	1
Racing	G52	control	A-G-A	A-G-G	1
Racing	G53	control	A-G-A	A-G-G	1
Racing	G54	case	T-A-G	A-G-G	0.997662
Racing	G55	control	A-G-A	A-G-G	1

Racing	G56	control	A-G-A	A-G-A	1
Racing	G57	control	A-G-A	A-G-A	1
Racing	G58	control	A-G-A	A-G-A	1
Racing	G59	control	A-G-A	A-G-G	1
Racing	G60	control	A-G-G	A-G-A	1
Racing	G61	control	A-G-A	A-G-A	1
Racing	G62	control	A-G-A	A-G-G	1
Racing	G63	case	A-G-A	A-G-A	1
Racing	G64	control	A-G-A	A-G-G	1
Racing	G65	control	A-G-A	T-G-A	1
Racing	G66	case	A-G-A	A-G-G	1
Racing	G67	control	A-G-A	A-G-A	0.575771
Racing	G68	case	A-G-A	A-G-G	1
Racing	G69	control	A-G-G	A-G-A	1
Racing	G70	control	A-G-A	A-G-A	1
Seppala	G71	control	A-G-A	A-G-A	1
Seppala	G72	control	A-G-A	A-G-A	1
Seppala	G73	control	A-G-G	A-G-A	1
Seppala	G74	control	A-G-A	A-G-A	1
Seppala	G75	control	A-G-A	A-G-A	1
Seppala	G76	control	A-G-G	A-G-A	1
Seppala	G77	control	A-G-A	A-G-A	1
Show	G78	control	A-G-A	A-G-A	1
Show	G79	control	A-G-A	A-G-A	1
Show	G80	control	A-G-A	A-G-A	1
Show	G81	control	A-G-A	A-G-A	1
Show	G82	control	A-G-A	A-G-A	1
Show	G83	control	A-G-A	A-G-A	1
Show	G84	control	A-G-A	A-G-A	1
Show	G85	control	A-G-A	A-G-A	1
Show	G86	case	T-A-G	A-G-A	0.997034

Show	G87	control	A-G-A	A-G-A	1
Show	G88	control	A-G-A	A-G-A	1
Show	G89	case	T-A-G	T-A-G	1
Show	G90	control	A-G-A	A-G-A	1
Show	G91	control	A-G-G	A-G-A	1
Show	G92	case	A-G-A	A-G-A	1
Show	G93	case	A-G-A	A-G-A	1
Show	G94	control	A-G-A	A-G-A	1
Show	G95	control	A-G-A	A-G-A	1
Show	G96	control	A-G-A	A-G-A	1
Show	G97	case	A-G-A	T-A-G	0.997034
Show	G98	case	T-A-G	T-A-G	1
Show	G99	control	A-G-A	A-G-A	1
Show	G100	control	A-G-A	A-G-A	1
Show	G101	control	A-G-A	A-G-A	1
Show	G102	case	A-G-A	T-A-G	0.997034
Show	G103	control	A-G-A	A-G-A	1
Show	G104	control	A-G-A	A-G-A	1
Show	G105	control	A-G-A	A-G-A	1
Sled-Show	G106	control	A-G-A	A-G-A	1
Sled-Show	G107	control	A-G-A	A-G-G	1
Sled-Show	G108	case	A-G-A	A-G-G	1
Sled-Show	G109	control	A-G-A	A-G-A	1
Sled-Show	G110	control	A-G-A	A-G-A	1
Sled-Show	G111	control	T-A-G	A-G-A	0.997034
Sled-Show	G112	control	A-G-A	A-G-A	1
Sled-Show	G113	control	A-G-A	A-G-A	1

Supplementary Table S2: Individual-level genotype assignments for the 73 Siberian Huskies that were Sanger sequenced. For each dog, the table reports population, subject ID, case/control status, and the genotype at the lead marker. Genotypes are reported in the *KCNIP4* (+) strand orientation. Accordingly, alleles appear as C/T in the Sanger-sequenced data and as the equivalent G/A calls in the SNP array data (strand-complemented representation).

Population	Sample ID	Status	Genotype BICF2P1282800
HuskyMix	S6	Case	CC
HuskyMix	G2 S9	Case	TT
Pet	G10 S10	Case	CT
Pet	G16 S13	Case	CT
Pet	S14	Case	CT
Pet	S24	Control	CC
Pet	G9 S25	Control	CC
Pet	S50	Control	CC
Pet	G3 S51	Control	CT
Pet	S52	Control	CT
Pet	S53	Control	CT
Pet	S54	Control	CC
Pet	S62	Case	TT
Pet	S69	Case	CC
Pet	S70	Control	CC
Pet	S77	Control	CC
Pet	S81	Case	CT
Pet	S82	Control	CT

Pet	S85	Case	CT
Racing	S2	Control	CC
Racing	G38 S16	Control	CC
Racing	S21	Control	CC
Racing	S22	Control	CC
Racing	G24 S23	Control	CC
Racing	S47	Case	CC
Racing	G44 S48	Case	CC
Racing	G34 S49	Case	CC
Racing	S84	Control	CC
Seppala	S46	Control	CC
Show	S1	Control	CC
Show	S7	Case	CT
Show	G102 S15	Case	CT
Show	G85 S17	Control	CC
Show	S18	Case	CT
Show	G81 S19	Control	CC
Show	S20	Control	CT
Show	S26	Case	CC
Show	S27	Case	CC
Show	S28	Control	CC
Show	S29	Control	CC
Show	S30	Control	CT

Show	G107 S31	Control	CC
Show	S32	Control	CC
Show	S33	Control	CC
Show	S35	Control	CC
Show	S37	Control	CC
Show	S38	Control	CC
Show	S39	Control	CC
Show	S40	Control	CT
Show	S41	Control	CC
Show	S42	Control	CC
Show	S43	Control	CT
Show	S44	Control	CC
Show	G113 S45	Control	CC
Show	G100 S57	Control	CC
Show	S58	Control	CC
Show	S59	Case	CC
Show	S60	Case	CC
Show	S61	Case	CC
Show	S64	Case	CT
Show	S66	Case	CC
Show	S67	Case	CC
Show	S68	Case	CT
Show	S71	Case	CT

Show	S72	Case	CT
Show	S73	Case	CT
Show	S74	Case	CC
Show	S83	Control	CC
Show	S87	Control	CT
Show	S88	Control	CT
Sled-Show	S12	Case	CC
Sled-Show	S76	Case	CC
Sled-Show	G108 S78	Case	CC

Supplementary Table S3. SNPs associated with idiopathic epilepsy in Siberian Huskies after Benjamini–Hochberg false discovery rate correction. *Results of GEMMA lmm genome-wide association testing for idiopathic epilepsy in Siberian Huskies, showing all SNPs with Benjamini–Hochberg FDR-adjusted q-values < 0.05, ordered by genomic position. For each SNP, chromosome (chr), SNP ID and nearest gene, position (CanFam3.1), effect allele (A1; reported as coded on the Illumina CanineHD array), effect allele frequency in cases and controls, the estimated per–effect-allele change in phenotype (beta), the standard error of beta (SE), Wald test p-value from the lmm, allelic odds ratio (OR) from PLINK, and FDR-adjusted q-value (FDR) are reported. SNPs exceeding the genome-wide Bonferroni-corrected significance threshold (p < 0.05 / 153704) are shown in* ***bold.***

chr	SNP/Gene	Position	A1	Freq Cases	Freq Controls	beta	se	P_Wald	OR	FDR
3	BICF2G630357319/*ADGRA3*	87335542	G	0.313	0.051	0.352	0.071	2.94E-06	8.5	0.021
3	BICF2G630357534/ Intergenic	87471234	A	0.354	0.073	0.323	0.064	1.92E-06	7.0	0.019
3	BICF2G630357943/*KCNIP4*	87829023	C	0.313	0.046	0.398	0.074	4.49E-07	9.5	0.006
**3**	**BICF2G630358500/ *KCNIP4***	**88292248**	**T**	**0.333**	**0.039**	**0.448**	**0.072**	**1.01E-08**	**12.2**	**2.38E-04**
3	TIGRP2P55899_rs8955313/ *KCNIP4*	88372659	G	0.396	0.096	0.322	0.062	9.47E-07	6.2	0.011
**3**	**BICF2P1282800/ *KCNIP4***	**88803651**	**A**	**0.354**	**0.017**	**0.531**	**0.071**	**1.90E-11**	**32.0**	**1.34E-06**
**3**	**BICF2S23653217/ *KCNIP4***	**88875038**	**G**	**0.604**	**0.227**	**0.312**	**0.050**	**8.80E-09**	**5.19**	**2.38E-04**
3	BICF2P305365/ *LOC111095408*	89784275	A	0.333	0.062	0.361	0.073	2.81E-06	7.59	0.021
16	BICF2G630115384/ Intergenic	34779818	A	0.208	0.022	0.488	0.099	2.89E-06	11.5	0.021
16	BICF2S23437988/*MICU3*	40403414	G	0.167	0.017	0.655	0.122	4.08E-07	11.7	0.006
18	BICF2P1218679/ *HECW1*	6680411	C	0.167	0.023	0.550	0.117	7.35E-06	8.6	0.043
X	chrX_95905113/ *TENM1*	95905113	A	0.156	0	0.510	0.107	5.40E-06	NA	0.035

Supplementary Table S4: Genome-wide association results for idiopathic epilepsy in the Show/Pet subset of Siberian Huskies. *Association testing was performed in GEMMA using a univariate lmm that included a genomic relatedness matrix to account for cryptic relatedness and the first two principal components (PC1–PC2) as fixed-effect covariates to control residual population structure. Analyses were restricted to the Show/Pet group (n = 58; 17 cases and 41 seizure-free controls). For each variant, the table reports the marker identifier (rs), genomic position (ps; CanFam3.1), effect allele (A1), reference allele (A0), estimated effect size (beta), standard error (se), and Wald test p-value. SNPs exceeding the genome-wide Bonferroni-corrected significance threshold (p < 0.05 / 153704) are shown in* ***bold.***

chr	rs	ps	A1	A0	af	beta	se	p_Wald
3	**BICF2S23653217**	**88875038**	**G**	**A**	**0.216**	**5.07E-01**	**7.95E-02**	**4.16E-08**
3	**BICF2P1282800**	**88803651**	**A**	**G**	**0.164**	**5.37E-01**	**8.74E-02**	**9.93E-08**
3	**BICF2G630358500**	**88292248**	**T**	**A**	**0.147**	**5.26E-01**	**8.95E-02**	**2.71E-07**
34	BICF2S23320040	8709729	G	A	0.207	4.44E-01	8.47E-02	2.76E-06
3	TIGRP2P55899_rs8955313	88372659	G	C	0.172	4.44E-01	8.57E-02	3.32E-06
3	BICF2G630357687	87532773	A	G	0.164	4.63E-01	9.24E-02	6.26E-06
3	BICF2G630357943	87829023	C	A	0.147	4.62E-01	9.59E-02	1.25E-05
3	BICF2G630357534	87471234	A	C	0.164	4.19E-01	9.01E-02	2.17E-05

Supplementary Table S5: Association of gonadal removal with epilepsy status and age-at-onset among CFA3 risk-allele carriers under two definitions (<5 years vs <2 years). OR= Odds ratio, HR= Hazard ratio, LR= Likelihood ratio test

**Outcome & model**	**Early threshold definition**	**Exposure definition (cases vs controls)**	**Early gonadectomy effect (95% CI)**	**p**	**Male effect (95% CI)**	**p**	**Overall model fit**
Epilepsy case status (Firth penalized logistic regression)	<5 years	Cases: exposed if gonadectomy < onset & 5y Controls: exposed if gonadectomy <5y	OR 5.98 (1.25–40.02)	0.024	OR 4.77 (1.05–25.76)	0.043	LR test p=0.0065
	<2 years	Cases: exposed if gonadectomy < onset & 2y Controls: exposed if gonadectomy <2y	OR 4.37 (0.92–27.69)	0.064	OR 4.12 (0.94–20.30)	0.060	LR test p=0.0149
Age at first seizure (Cox PH with time-varying exposure)	<5 years	Dogs contribute unexposed time from birth→ gonadectomy; exposed time begins after gonadectomy only if gonadectomy <5y and prior to endpoint[†]	HR 2.69 (1.08–6.73)	0.034	HR 3.12 (1.33–7.32)	0.0089	Concordance 0.68; Schoenfeld global p=0.176
	<2 years	As above, with exposure beginning only if gonadectomy <2y and prior to endpoint[†]	HR 2.35 (0.97–5.71)	0.0595	HR 3.04 (1.26–7.34)	0.0136	Concordance 0.676; Schoenfeld global p=0.36

[†] *Endpoint was seizure onset age for cases and age at last follow-up for controls; controls were restricted to ≥7.5 years at last follow-up.*

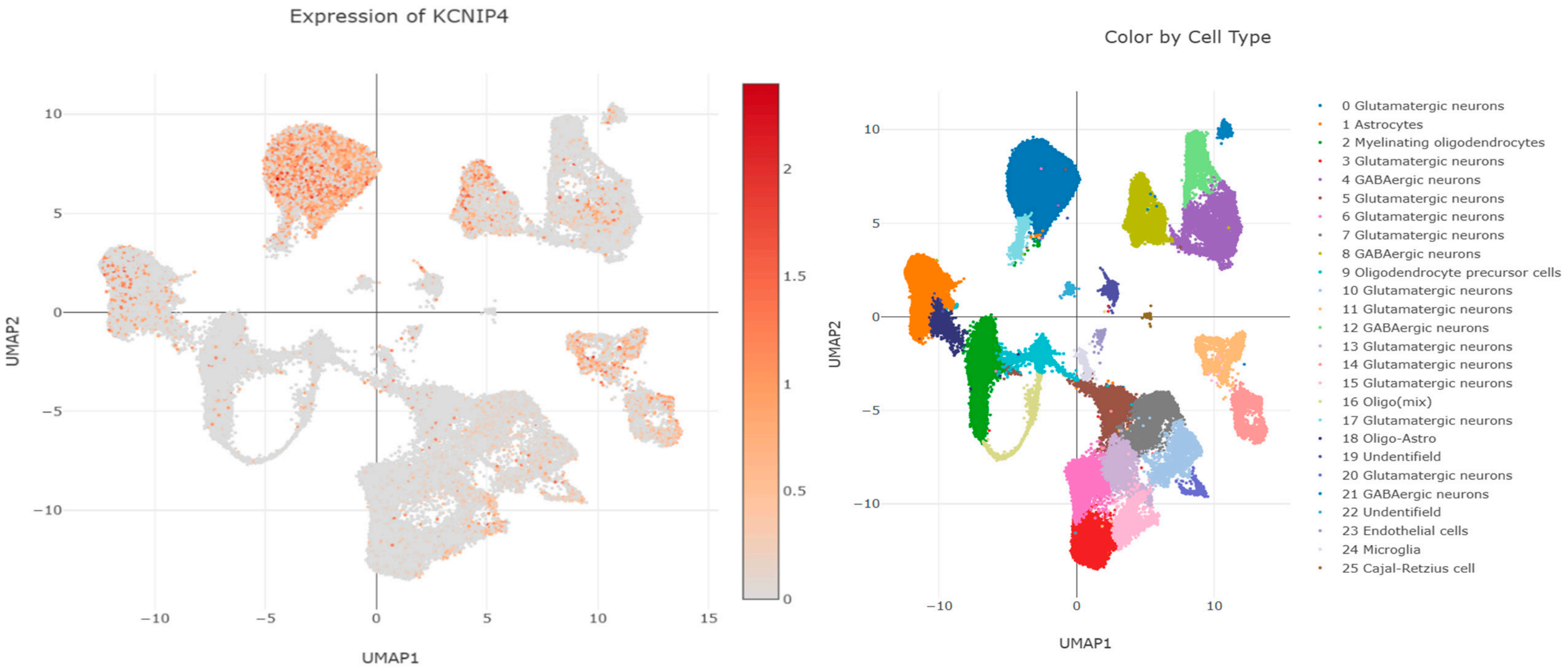

Supplementary Figure S1: Single-nucleus expression of *KCNIP4* in the canine hippocampus. UMAP projection of a published single-nucleus RNA-seq atlas generated from hippocampi of 5-month-old Beagle dog from https://dog10k.kiz.ac.cn/SnRNA (Zhou et al., 2022; Zhou et al., 2024). Each point represents one nucleus positioned by transcriptomic similarity. Points are colored by *KCNIP4* expression level (gray = not detected/low expression; orange/red = higher expression), illustrating that *KCNIP4* transcripts are concentrated in glutamatergic and GABAergic neurons.

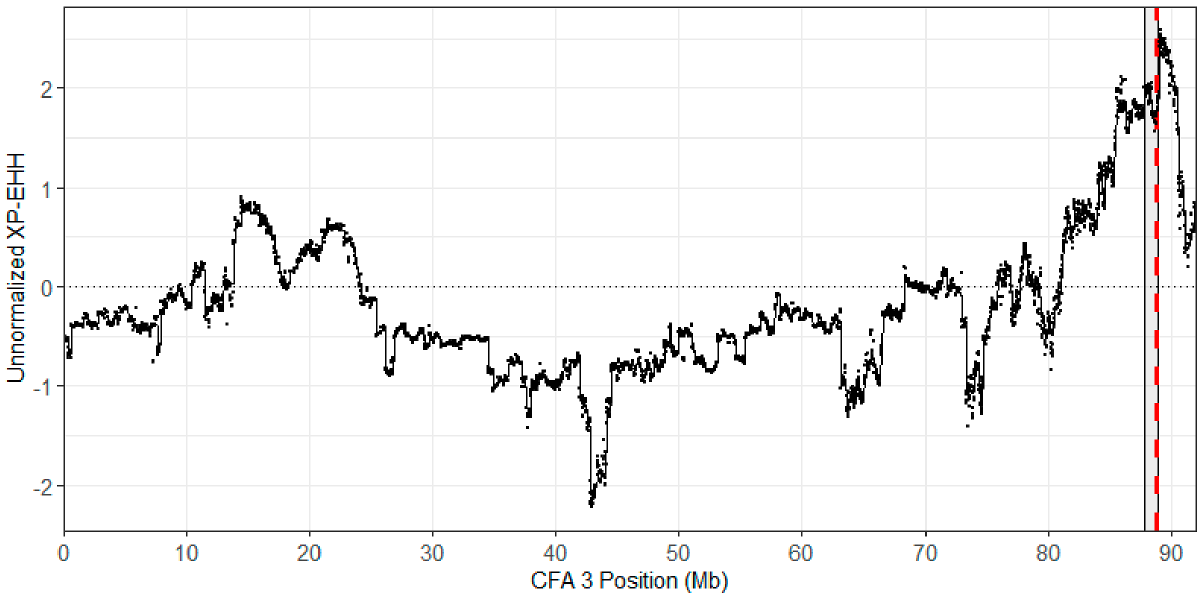

Supplementary Figure S2: Unnormalized XP-EHH across CFA3 in the Show/Pet group. XP-EHH was computed as log(iES_Risk / iES_NonRisk) from phased haplotypes, where risk and non-risk chromosomes were defined by the allele carried at the lead risk marker, BICF2P1282800 (chr3:88,803,651; CanFam3.1). Points show per-marker XP-EHH values, and the solid curve shows a running-median smooth. The horizontal dotted line at 0 indicates no difference in haplotype homozygosity between backgrounds; positive values indicate longer, more homogeneous haplotypes on risk chromosomes. The red dashed vertical line marks the position of BICF2P1282800, and the shaded box denotes *KCNIP4*.

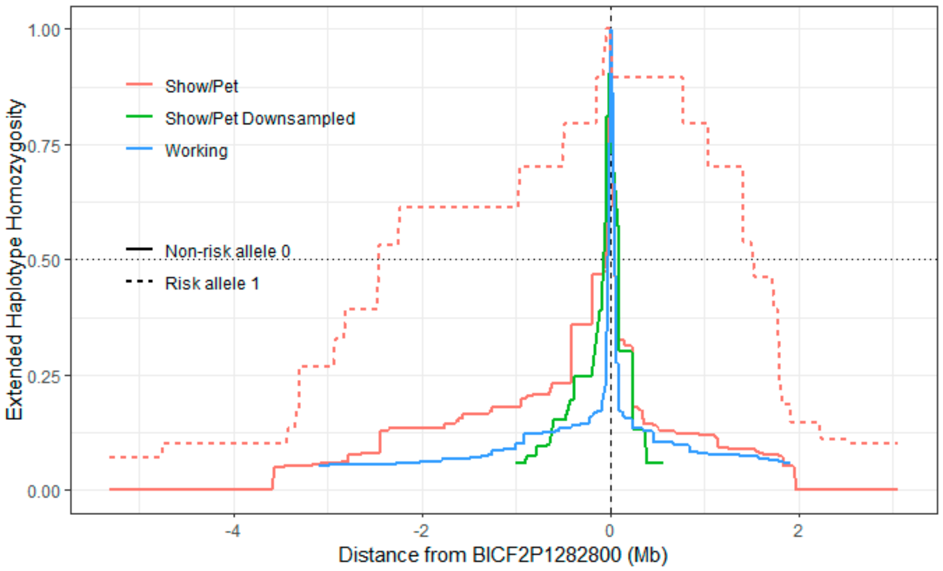

Supplementary Figure S3: Extended haplotype homozygosity (EHH) decay centered on the lead risk marker. EHH was computed for haplotypes carrying the non-risk (solid lines) and risk (dashed lines) alleles at the lead risk marker to measure how rapidly homozygosity declines with physical distance from the lead marker on the different backgrounds. The vertical dashed line marks the lead risk marker (0 Mb) and the horizontal dotted line indicates half-decay, EHH = 0.5. Curves are shown for the Show/Pet subset (red) and Working subset (blue). To assess whether unequal sample size could account for differences in decay, a Show/Pet down-sampled analysis (green) was generated by randomly selecting 10 Show/Pet non-risk controls and recomputing EHH using the same pipeline.

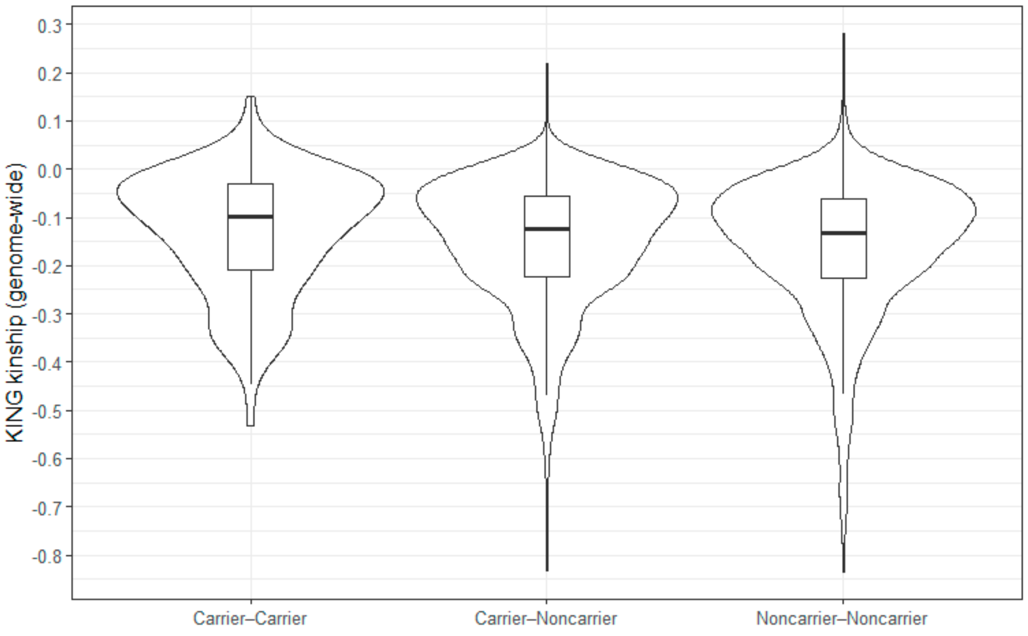

Supplementary Figure S4: Genome-wide relatedness by CFA3 risk-marker carrier status. Violin plots show the distribution of genome-wide KING-robust kinship coefficients for all unique dog pairs classified as carrier–carrier, carrier–noncarrier, or noncarrier–noncarrier based on risk allele dosage at the lead CFA3 marker (BICF2P1282800; carriers defined as dosage ≥ 1). Embedded boxplots summarize the median (horizontal line) and interquartile range (box), with whiskers indicating the spread of the central distribution. Kinship was computed from LD-pruned autosomal SNPs, and more negative values indicate lower relatedness relative to the sample's overall allele-frequency baseline.

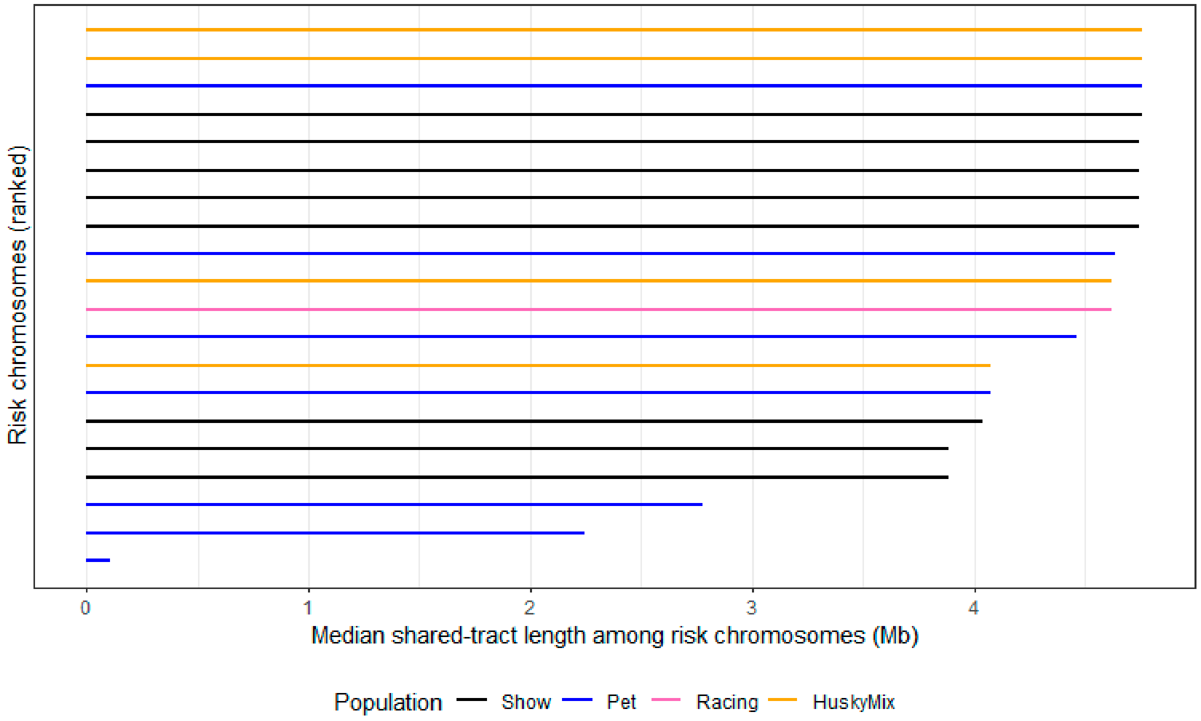

Supplementary figure S5: Median shared-tract length among risk chromosomes by subpopulation. Each horizontal segment represents a single phased chromosome (haplotype), ranked by length within panel, and the x-axis shows the median shared haplotype-tract length (Mb) with all other chromosomes carrying the same allele, computed from pairwise shared-tract lengths extending on both sides of the lead marker.
